# Effects of Mulching on Soil Properties and Growth of Tea Olive (*Osmanthus fragrans*)

**DOI:** 10.1371/journal.pone.0158228

**Published:** 2016-08-10

**Authors:** Xue Ni, Weiting Song, Huanchao Zhang, Xiulian Yang, Lianggui Wang

**Affiliations:** 1 College of Landscape Architecture, Nanjing Forestry University, Nanjing, 210037, PR China; 2 College of Civil Engineering and Architecture, Anhui University of Technology, Maanshan, 243000, PR China; 3 College of Forestry, Nanjing Forestry University, Nanjing, 210037, PR China; DOE Pacific Northwest National Laboratory, UNITED STATES

## Abstract

Different mulches have variable effects on soil physical properties and plant growth. This study aimed to compare the effects of mulching with inorganic (round gravel, RG), organic (wood chips, WC), and living (manila turf grass, MG) materials on soil properties at 0–5-cm and 5–10-cm depths, as well as on the growth and physiological features of *Osmanthus fragrans* L. ‘Rixianggui’ plants. Soil samples were collected at three different time points from field plots of *O*. *fragrans* plants treated with the different mulching treatments. Moisture at both soil depths was significantly higher after mulching with RG and WC than that in the unmulched control (CK) treatment. Mulching did not affect soil bulk density, pH, or total nitrogen content, but consistently improved soil organic matter. The available nitrogen in the soil increased after RG and WC treatments, but decreased after MG treatment during the experimental period. Mulching improved plant growth by increasing root activity, soluble sugar, and chlorophyll a content, as well as by providing suitable moisture conditions and nutrients in the root zone. Plant height and trunk diameter were remarkably increased after mulching, especially with RG and WC. However, while MG improved plant growth at the beginning of the treatment, the ‘Rixianggui’ plants later showed no improvement in growth. This was probably because MG competed with the plants for water and available nitrogen in the soil. Thus, our findings suggest that RG and WC, but not MG, improved the soil environment and the growth of ‘Rixianggui’ plants. Considering the effect of mulching on soil properties and plant growth and physiology, round gravel and wood chips appear to be a better choice than manila turf grass in ‘Rixianggui’ nurseries. Further studies are required to determine the effects of mulch quality and mulch-layer thickness on shoot and root growths.

## Introduction

Since the late 1930s, mulching has been used for the environmental modification of forests, agriculture lands, and urban landscapes [1]. This process has many advantages: mulches are known to buffer soil temperature [2], prevent soil water loss by evaporation [3], inhibit weed germination, and suppress weed growth [4]. Further, they can protect soils from wind-, water-, and traffic-induced erosion and compaction [1]. Finally, mulch can improve crop production by enhancing soil quality by conserving soil moisture, enhancing soil biological activities, and improving the chemical and physical properties of soil [5,6]. Thus, mulching in urban or ornamental landscapes improves not only soil quality, but also plant growth.

Landscape mulches are generally inorganic (gravel, pebbles, or polyethylene film) [7], organic (wood, bark, or leaves, used individually or in mixtures), or living (turf grass, rye, and clover) materials. In developing countries, gravel is usually preferred because of its low cost and wide availability. Gravel effectively reduces evaporation and runoff, improves infiltration, moderates soil temperature, and maintains soil fertility [8]. It can also indirectly improve crop yield via the interaction between increased soil water and moderated soil temperature [9]. Wood-based mulches are commonly used to improve the appearance of landscapes [10]. They also conserve soil moisture; reduce weed invasion and soil temperature fluctuations; and they improve plant growth, yield, and quality [10–12]. Although living mulches require soil water, they can reduce surface temperatures by releasing water vapor via evapotranspiration [13]. Moreover, living mulches decompose faster under appropriate water and temperature conditions and release nutrients into the soil that can be used by plants and microbes. However, the effects of mulches and their extent depend on the mulch type, soil chemistry, and the importance of the released nutrients [1].

While there are many benefits to using mulches, they can also damage soil quality and decrease plant growth. Inorganic mulches made from rock, gravel, and crushed brick can increase temperatures above and below the mulch layer and cause soil alkalinization, resulting in injuries to plant stems [14]. Wood-based mulches also have several limitations, including temporary soil nitrogen deficiency [15], potential fire hazard [16], and increased risk of introducing exotic plant pathogens to urban landscapes from the uncomposted wood chips [10]. Living mulches often compete for nutrients and water, especially on landscapes with relatively high soil fertility. In addition, allelopathic effects of cool-season turf grasses on woody plants can inhibit tree growth. [17]. Very few studies have compared the effects of inorganic, organic, and living mulches on soil quality and plant growth [14,18].

*Osmanthus fragrans* Lour. ‘Rixianggui’, a member of the family Oleaceae [19], is widely distributed and cultivated as an ornamental plant in southern and central China, where it is considered as one of the most popular traditional flowers [20]. This study aimed to compare the effects of three types of mulches (inorganic, gravel; organic, wood chips; and living, manila turf grass) on soil properties, plant growth, and the physiological performance of *O*. *fragrans*.

## Materials and Methods

### Site description

Field experiments were conducted between April 2013 and June 2014 in a nursery (31° 57ʹ 39″ N, 119° 12ʹ 25″ E) at the Institute of Landscape Architecture, Nanjing Forestry University, China. The subtropical location of the nursery is characterized by its humid climate with an annual mean temperature and precipitation of 15.2°C and 1,012 mm, respectively. The soil in the experimental field is classified as yellow brown soil (25.6% clay, 68.8% silt, and 5.6% sand) and was cleared and hoed manually before the onset of the experiments.

### Experimental design

Four treatments were established in 6 m × 2 m plots: (1) unmulched control soil (CK); (2) inorganic mulch, approximately 1.5 cm layer of <4 cm diameter rounded gravel (RG; 1250 t·ha^−1^); (3) organic mulch (WC), an approximated 1 cm layer of wood chips that extended 3–4-cm in length from dried mature *Pinus squamata* X. W. Li (127.5 t·ha^−1^); and (4) living mulch (MG), a 5-cm layer of 25 cm × 25 cm pieces of manila turf grass with soil and roots attached. *O*. *fragrans* were first grown to approximately 30 cm in height in a greenhouse and then transplanted to the plots at a density of 12 plants per plot (one plant per square meter). The mulches were applied after all *O*. *fragrans* had been planted. The experiment was a randomized complete block design with three replicates.

During the experimental period, no fertilizer was applied and watering and weeding practices were consistent with those used by local farmers. After the seedlings were transplanted, roots and leaves were pump-irrigated once each day during the first three days and then irrigated once a week in the first month. Plants were irrigated every 15 days at 04:00 pm during the summer, but no irrigation was performed during the winter. Weeds were removed manually every 20–30 days between April and November.

### Soil sampling and analysis

Soils were sampled at three different times: May 23, 2013; October 23, 2013; and May 23, 2014. Before soil core sampling, mulch was removed from the sampling area to prevent the contamination of the cores with surface organic matter. Approximately 20 soil cores were randomly collected from each plot and divided into two layers: 0–5 cm and 5–10 cm. In the field, each sample was divided into two parts and sealed in plastic bags: one part was stored at 4°C for the analysis of basic soil properties; the other part was air-dried in a ventilated room, ground, and filtered through a < 2-mm mesh to remove stones, root fragments, and organic debris before performing chemical analyses.

Soil samples that had been air-dried and filtered through a 2-mm mesh were used to determine the available nitrogen (N) content and measure pH. Additional samples were passed through a 0.149-mm mesh to estimate organic matter and total N contents. Soil organic matter (SOM) was measured using H_2_SO_4_–K_2_Cr_2_O_7_ wet oxidation, followed by titration with FeSO_4_ according with the Walkley–Black procedure [21]; soil total nitrogen (STN) was determined using micro-Kjeldahl digestion, followed by colorimetric analysis [22, 23]. Soil pH was measured in a 1: 2.5 (m/v) soil: water ratio by using a pHS-3C pH/mV meter (Rex Ltd., Shanghai, China). Soil moisture was determined after the soil core samples were oven-dried at 105°C for 8 h [24]. Soil available nitrogen (SAN) was determined using the alkali-hydrolytic diffusion method [25]. Soil bulk density was measured from samples obtained using a volumetric steel ring (100 cm^3^) and calculated as the mass of oven-dried soil (105°C), divided by the core volume for each measurement depth.

### Plant growth and physiological features

Trunk diameter and plant height were determined on the same days soil were sampled; diameter was measured at 15 cm above the soil surface using a caliper, and height was measured from the soil surface to the highest point in the tree crown. Simultaneously, roots and leaves were collected to determine root activity and the relative water content (RWC); relative electric conductivity (REC); and chlorophyll, soluble sugar, and free proline content of the leaves. Root activity was measured using the triphenyl tetrazolium chloride (TTC) method [26]; RWC, using Barrs and Weatherley’s method [27, 28]; and REC, which indicates the permeability of a leaf, was measured with a DDS-11A meter (Rex Ltd., Shanghai, China) [29]. Chlorophyll content was measured spectrophotometrically by the method and equations proposed by Lorenzen [30]. Leaf soluble sugar and proline contents were quantified in extracts of fresh leaves (0.1 g) in potassium phosphate buffer (50 mM, pH = 7.5) [31]. The extracts were filtered through four layers of cheesecloth and centrifuged at 15,500 rpm for 15 min at 4°C, and the resulting supernatant was collected and stored at 4°C. Soluble sugar was analyzed using the anthrone reagent and a Bausch and Lomb spectrophotometer [32]. Free proline was estimated by spectrophotometric analysis of a ninhydrin reaction solution [33] at 515 nm in a UV-2900 spectrophotometer (HITACHI, Japan).

### Statistical analysis

All data were subjected to analysis of variance (ANOVA) tests, and the means were compared using Student’s *t*-tests by using JMP version 9.0 (SAS Institute Inc., Cary, NC, USA). A repeated measures ANOVA was performed for soil properties, plant height, and trunk diameter to analyze the effects of mulch type and sampling time. Differences were considered significant at *P* < 0.05.

## Results and Discussion

### Mulching materials have differential effects on soil properties

Soil properties showed varying effects over time to the different mulching treatments. The average soil moisture at the 0–5-cm depth layer of CK, RG, WC, and MG plots was 18.0%, 20.3%, 21.6%, and 20.3%, respectively (Table 1). Similarly, the average soil moisture at the 5–10-cm depth was 19.2%, 21.7%, 21.6%, and 20.6%, respectively (Table 2). Soil moisture values were higher in June 2014 than in May 2013 in the top and bottom layers of all treatments, indicating that mulching increases soil moisture. WC treatment had a stronger effect on soil moisture than the RG and MG treatments (Tables 1 and 2).

**Table 1:** Soil properties at the 0–5 cm depth at the three sampling time points with and without mulching treatments.

Sampling time	Treatment	Moisture (%)	Bulk density (g·cm^−3^)	pH	STN (g·kg^−1^)	SAN (g·kg^−1^)	SOM (g·kg^−1^)	C/N
2013-5-23	CK	18.6 ± 1.7b	1.33 ± 0.05a	5.8 ± 0.2bc	0.58 ± 0.09a	50.2 ± 4.7a	8.4 ± 1.5b	8.4 ± 0.7a
	RG	21.3 ± 1.8ab	1.37 ± 0.06a	6.1 ± 0.1a	0.56 ± 0.03a	54.5 ± 2.6 a	10.4 ± 1.5ab	10.8 ± 1.9a
	WC	24.3 ± 0.3a	1.32 ± 0.08a	6.0 ± 0.1ab	0.56 ± 0.05a	56.1 ± 1.7a	12.9 ± 1.8a	13.5 ± 2.7a
	MG	20.5 ± 2.4ab	1.35 ± 0.07a	5.7 ± 0.2c	0.56 ± 0.05a	52.8 ± 5.6 a	10.8 ± 1.1ab	11.4 ± 2.0a
2013-10-23	CK	14.2 ± 0.6b	1.30 ± 0.05a	5.8 ± 0.2a	0.55 ± 0.07a	34.8 ± 7.0ab	8.3 ± 1.2c	8.8 ± 1.0b
	RG	16.6 ± 1.4a	1.35 ± 0.06a	6.0 ± 0.1a	0.50 ± 0.05a	39.1 ± 4.5ab	11.5 ± 0.9b	13.6 ± 2.5a
	WC	16.0 ± 0.5a	1.29 ± 0.08a	5.9 ± 0.2a	0.48 ± 0.07a	43.9 ± 5.5a	13.6 ± 0.5a	15.1 ± 0.5a
	MG	15.8 ± 0.6a	1.29 ± 0.10a	5.8 ± 0.2a	0.51 ± 0.03a	28.4 ± 5.9b	11.1 ± 1.2b	12.5 ± 1.7a
2014-6-23	CK	21.3 ± 1.5b	1.32 ± 0.08a	5.9 ± 0.1a	0.55 ± 0.13a	23.1 **±** 1.1b	8.5 ± 1.4c	9.3 ± 2.2b
	RG	22.9 ± 1.3ab	1.33 ± 0.07a	5.9 ± 0.1a	0.50 ± 0.03a	29.9 ± 1.0a	9.8 ± 0.6bc	10.9 ± 1.6b
	WC	24.6 ± 1.9a	1.34 ± 0.02a	5.8 ± 0.1a	0.51 ± 0.10a	31.7 ± 0.7a	13.8 ± 1.1a	14.2 ± 1.3a
	MG	24.6 ± 1.8a	1.32 ± 0.02a	5.9 ± 0.1a	0.53 ± 0.07a	21.8 ± 1.2b	11.3 ± 0.9b	12.4 ± 1.5ab

No mulching (CK); mulching with round gravel (RG), wood chips (WC),or manila turf grass (MG). STN: soil total nitrogen; SOM: soil organic matter; SAN: soil available nitrogen; C/N: carbon to nitrogen ratio. Values with different letters in the same column indicate significant differences between treatments (*P* < 0.05, n = 3). Data are means ± standard deviation.

**Table 2:** Soil properties at the 5–10-cm depth at the three sampling time points with and without mulching treatments.

Sampling time	Treatment	Moisture (%)	Bulk density (g·cm^−3^)	pH	STN (g·kg^−1^)	SAN (g·kg^−1^)	SOM (g·kg^−1^)	C/N
2013-5-23	CK	20.4 ± 3.3b	1.32 ± 0.08a	5.7 ± 0.2a	0.57 ± 0.04a	50.4 ± 5.1a	9.3 ± 0.8b	9.5 ± 1.0b
	RG	23.2 ± 2.0ab	1.38 ± 0.07a	5.8 ± 0.1a	0.56 ± 0.06a	54.8 ± 2.8a	11.3 ± 0.9a	11.7 ± 0.3ab
	WC	23.1 ± 0.4a	1.31 ± 0.06a	5.7 ± 0.1a	0.52 ± 0.03a	53.2 ± 4.1a	11.8 ± 1.3a	13.2 ± 1.9a
	MG	21.4 ± 2.6b	1.36 ± 0.03a	5.7 ± 0.1a	0.55 ± 0.07a	54.6 ± 4.7a	11.3 ± 0.7a	12.1 ± 1.3a
2013-10-23	CK	14.8 ± 1.2b	1.31 ± 0.02a	5.9 ± 0.0a	0.60 ± 0.07a	34.1 ± 5.1ab	8.4 ± 1.3b	8.2 ± 1.5b
	RG	17.4 ± 0.8a	1.33 ± 0.10a	6.0 ± 0.1a	0.52 ± 0.04a	39.3 ± 2.6a	10.8 ± 1.0a	12.1 ± 0.6a
	WC	17.3 ± 1.4a	1.31 ± 0.05a	5.9 ± 0.2a	0.49 ± 0.08a	40.8± 2.5a	11.4 ± 0.7a	14.6 ± 2.3a
	MG	17.7 ± 1.0a	1.35 ± 0.05a	5.8 ± 0.1a	0.54 ± 0.07a	31.2 ±5.6b	10.5 ± 0.8a	11.4 ± 2.1ab
2014-6-23	CK	22.4 ± 0.9b	1.33 ± 0.05a	5.9 ± 0.1a	0.52 ± 0.04a	23.1 ± 1.8b	8.6 ± 0.5b	9.7 ± 0.4c
	RG	24.3 ± 1.5ab	1.36 ± 0.09a	5.9 ± 0.1a	0.47 ± 0.08a	29.9 ± 1.4a	11.8 ± 1.2a	14.7 ± 1.5ab
	WC	24.5 ± 0.6a	1.37 ± 0.06a	5.8 ± 0.0a	0.45 ± 0.05a	30.2 ± 1.9a	12.7 ± 1.9a	15.0 ± 0.9a
	MG	22.7 ± 1.0ab	1.36 ± 0.08a	6.0 ± 0.1a	0.53 ± 0.07a	20.3 ± 1.8b	10.5 ± 1.2ab	12.7 ± 1.4bc

No mulching (CK); mulching with round gravel (RG), wood chips (WC), or manila turf grass (MG). STN: soil total nitrogen; SOM: soil organic matter; SAN: soil available nitrogen; C/N: carbon to nitrogen ratio. Values with different letters in the same column indicate significant differences between treatments (*P* < 0.05, n = 3). Data are means ± standard deviation.

All of mulch types had significant effects on soil moisture in both soil layers measured except for MG at the 5–10-cm depth (Table 3). These results are consistent with previous studies that suggest mulching with gravel [14, 34, 35], wood chips [2,3, 11, 12, 14], and grass [36] sequesters water and prevents water loss from the soil through evaporation; additionally, organic mulches conserve water more effectively than inorganic ones [14,37]. Adequate water is essential for plant growth. However, some studies show that living mulches might compete with plants for water and hence, mulched soils can show lower moisture content than bare soils [11]. In the present study, mulching with turf grass significantly increased soil moisture at the 0–5-cm depth, but had no effect on soil moisture at the 5–10-cm depth (Table 3). These results may be influenced by the high precipitation at the study site.

**Table 3:** Significance (*P* < 0.05) of soil properties, plant height, and trunk diameter at the two soil depths (0–5 cm and 5–10 cm) over time after the three mulching treatments assessed by ANOVA of three replicates.

Parameters	Moisture		pH		SOC		C/N		Available N		Plant height	Trunk diameter
	0−5 cm	5−10 cm	0−5 cm	5−10 cm	0−5 cm	5−10 cm	0−5 cm	5−10 cm	0−5 cm	5−10 cm		
RG	*0*.*006*	*0*.*015*	*0*.*003*	0.142	*<0*.*001*	*<0*.*001*	*0*.*004*	0.142	*0*.*020*	*0*.*006*	*<0*.*001*	*<0*.*001*
Time	*<0*.*001*	*<0*.*001*	0.774	*0*.*033*	0.921	0.631	0.310	*0*.*033*	*<0*.*001*	*<0*.*001*	*<0*.*001*	*<0*.*001*
RG*Time	0.807	0.907	0.292	0.545	0.951	0.740	0.303	0.545	0.829	0.828	0.521	0.179
WC	*<0*.*001*	*0*.*008*	0.252	0.628	*<0*.*001*	*<0*.*001*	*<0*.*001*	0.628	*0*.*002*	*0*.*008*	*<0*.*001*	*<0*.*001*
Time	*<0*.*001*	*<0*.*001*	0.817	0.113	0.789	0.803	0.534	0.114	*<0*.*001*	*<0*.*001*	*<0*.*001*	*<0*.*001*
WC*Time	0.066	0.936	0.412	0.675	0.833	0.904	0.724	0.675	0.785	0.547	0.291	*0*.*006*
MG	*<0*.*001*	0.149	0.836	0.964	*0*.*004*	*0*.*004*	*0*.*001*	0.964	0.468	0.807	*0*.*004*	*0*.*001*
Time	*<0*.*001*	*<0*.*001*	0.368	*0*.*021*	0.460	0.302	0.555	*0*.*021*	*<0*.*001*	*<0*.*001*	*<0*.*001*	*<0*.*001*
MG*Time	0.617	0.483	0.738	0.548	0.321	0.236	0.920	0.548	0.307	0.315	*0*.*010*	0.682

RG, round gravel; WC, wood chips; MG, manila turf grass; SOC, soil organic carbon; C/N, carbon to nitrogen ratio. Significant values are italicized.

Mulching had no effect on the bulk density of either soil layer (Tables 1 and 2). At the 0–5-cm depth, soil pH was 5.2% higher in the RG treatment compared to the CK treatment (Table 1); no significant change in soil pH was noted in the other treatments. However, pH values in the RG treatment did not change in the following sampling times (Tables 1 and 2), indicating that the effect was ephemeral. The elevated pH values possibly resulted from the leaching of basic cations (NH_4_^+^) from the decomposing SOM [38]. At the 5–10-cm depth, no significant differences in soil pH were observed among treatments. Billeaud and Zajicek [39] reported that mulching with four types of organic mulches (screened pine bark, hardwood, cypress, and decorative pine bark nuggets) significantly decrease soil pH in a soil composed of fine sandy loam. Duryea et al. [18] also found that pine bark mulches decrease soil pH. However, Iles and Dosmann [14] found that mulching with inorganic (e.g., river rock and lava rock) and organic (e.g., wood chips and shredded bark) mulches remarkably increased soil pH in a Nicollet fine sandy loam soil. Taken together with our findings, these results suggest that the effect of mulches on soil pH depends on the mulching material as well as soil composition/type.

The three mulching treatments analyzed in this study did not affect STN content at any soil depth during the experimental period (Tables 1 and 2). At the first and second sampling times, no obvious differences were found in the SAN content between mulched soils and bare soil at both the sampling depths. Nonetheless, WC and MG treatments increased SAN contents by 29.4% and 37.2% at the 0–5-cm depth, and by 29.4% and 30.7% at the 5–10-cm depth, respectively, at the third sampling time (Tables 1 and 2). Repeated measures ANOVA showed that RG and WC treatments significantly altered SAN content, whereas the MG treatment did not (Table 3). Since organic mulches decompose under appropriate water and temperature levels, nutrients are released to the soil and become available for root uptake or microbial use [1]. Although not significantly, SAN contents decreased in the MG treatment over time (Tables 1 and 2); this may be mainly attributed to the competition for nutrients between turf grass and plants [1]. Gravel mulches always contain fewer nutrients and are difficult for microorganisms to decompose. Thus, the increase in SAN content after RG treatment may be because the gravel provided suitable conditions for the growth of microorganisms that released more nutrients via SOM decomposition. In addition, gravel mulch can trap dirt, which contains nitrogen and organic matter; therefore, nutrient content of gravel-mulched fields is high [8]. The significant decrease in SAN contents over time can be caused by plant nutrient uptake.

SOM is derived from the decay of dead organisms and consists of organic (carbon-based) compounds [40]. It positively contributes to tree and environmental health through its effects on soil physical, chemical, and biological properties [41]. Mulches increased SOM content and the ratio of carbon (C) to N at both soil depths tested (Tables 1 and 2) and repeated measures ANOVA also showed that the three mulching treatments significantly altered SOM content at both soil depths (*P* < 0.05). Although the three treatments had significant effects on the ratios of C to N at the 0–5-cm depth, they had no significant effect on these ratios at the 5–10-cm depth (Table 3). Previous studies have shown that organic materials increase SOM by directly improving soil properties [42], increasing photosynthesis, and by having an impact on belowground C allocation [43]. Manila turf grass remarkably influences soil properties and processes, including the increase in SOM [44, 45]. The stimulation of plant growth after the three mulching treatments can be attributed to an increase in photosynthesis, resulting in the higher sequestration of C.

### Mulching materials varyingly affect plant physiological features

Several plant physiological features were measured to evaluate the effects of the mulches on plant health. Although root activity is an important physiological parameter for evaluating ion uptake, few studies have considered how it is affected by mulch treatment. Root activity as measured by TTC reducing capacity, was highest in WC, followed by RG, MG, and CK (Table 4). Moreover, root activity significantly increased after WC, RG, and MG treatments, suggesting that plants grown in mulched soils take up more nutrients than those grown in unmulched soils. Thus, the present findings are consistent with those of Chalker-Scott [1] who showed that root development and density are greater in soils treated with organic mulches than in those treated with nothing or plastic or living mulches.

**Table 4:** Physiological features of plants measured on May 23, 2014 in bare soil (CK) or soils treated with round gravel (RG), wood chips (WC), and manila turf grass (MG).

Treatment	Root activity	RWC	REC	Proline	Soluble sugar	Chlorophyll a	Chlorophyll b	Chlorophyll
	(μg·g^−1^·h^−1^)	(%)	(%)	(μg·g^−1^)	(%)	(mg·g^-1^)	(mg·g^-1^)	(mg·g^-1^)
CK	232.1 ± 9.0cb	82.9 ± 2.8a	34.5 ± 5.1a	0.10 ± 0.00a	0.64 ± 0.09b	0.80 ± 0.10c	0.60 ± 0.04a	1.39 ± 0.11c
RG	280.1 ± 9.7a	83.3 ± 2.6a	33.8 ± 8.9a	0.12 ± 0.02a	0.88 ± 0.04a	1.09 ± 0.08ab	0.51 ± 0.04a	1.60 ± 0.12ab
WC	289.0 ± 12.6a	83.7 ± 2.4a	41.0 ± 4.3a	0.08 ± 0.02a	0.86 ± 0.11a	1.17 ± 0.10a	0.57 ± 0.03a	1.74 ± 0.13a
MT	253.4 ± 11.3b	83.5 ± 3.2a	38.5 ± 6.8a	0.09 ± 0.01a	0.79 ± 0.06a	0.97 ± 0.02b	0.51 ± 0.06a	1.48 ± 0.07bc

RWC, relative water content of leaves; REC, relative electric conductivity; chlorophyll is the sum of chlorophyll a and chlorophyll b. Different letters in the same column indicate significant differences between treatments (*P* < 0.05, n = 3). Data are means ± standard deviation.

The different mulches did not affect the RWC, REC, and proline content of leaves. However, the soluble sugar content increased by 37.5%, 34.4%, and 23.4% in RG, WC, and MG treatments, respectively, suggesting that mulches stimulated stress resistance of plants. Mulches did not change the chlorophyll b content, although chlorophyll a content increased by 36.3%, 46.3%, and 21.3% in the RG, WC, and MG treatments, respectively, indicating that mulches enhanced the photosynthetic rate in the leaves of plants grown under these conditions.

Some studies have shown that soil mulching decreases plant health by increasing plant stem temperature [14] and causing soil alkalinization or acidification [11, 14]. In addition, uncomposted wood chips derived from wood packing materials can increase the risk of introducing exotic plant pathogens to urban landscapes [10]. Conversely, the findings of this study suggest that mulching with round gravel, wood chips, and manila turf grass positively affect plant physiological features, thus benefiting the health and growth of ‘Rixianggui’.

### Mulches differentially affect plant height and trunk diameter

**Fig 1:**
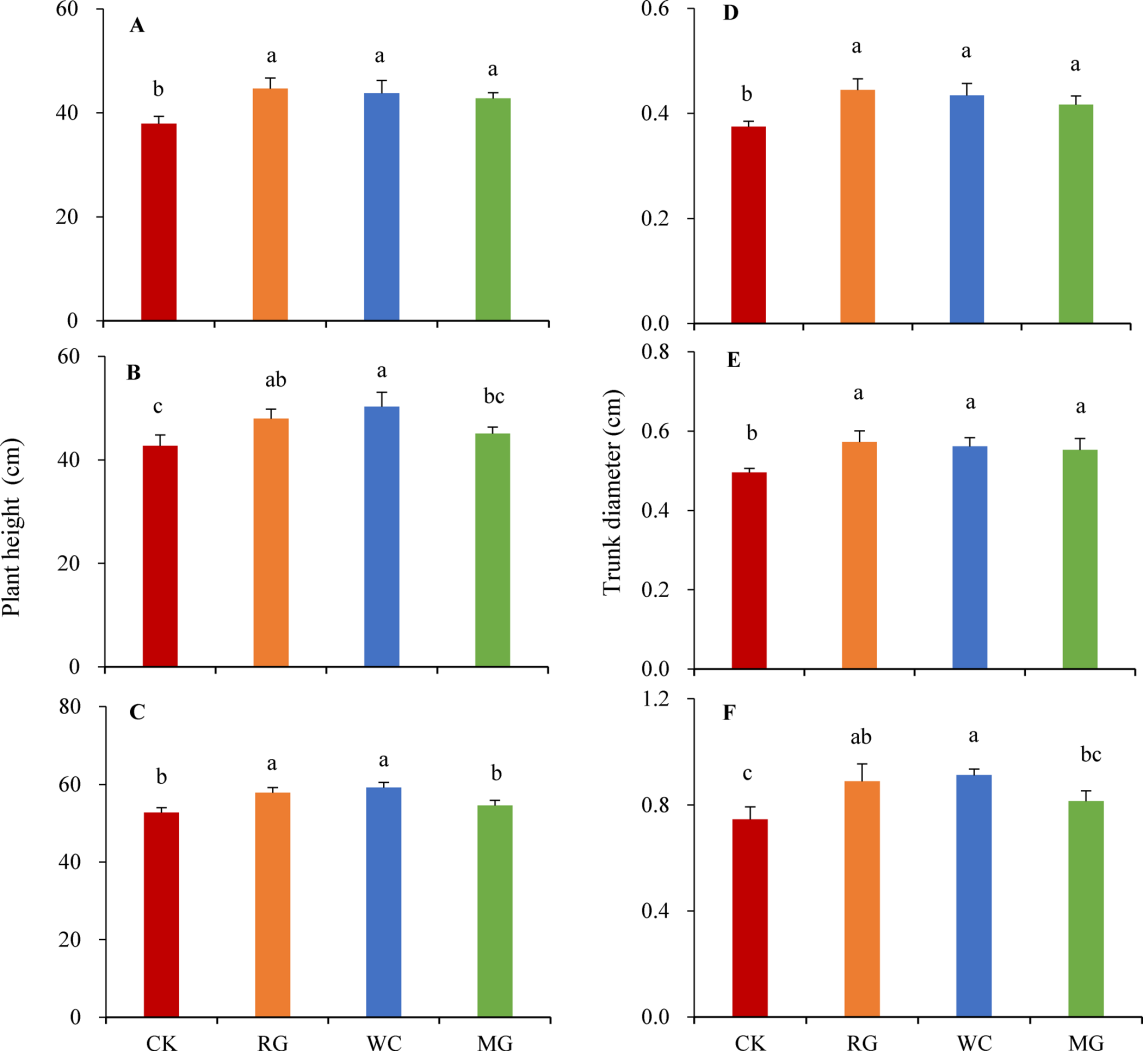
Effects on plant height and trunk diameter according to mulch type. The physiological parameters of plants grown in unmulched soil (CK) and those grown in soil mulched with round gravel (RG), wood chips (WC), and manila turf grass (MG) were measured on May 23, 2013 (**A, D**); October 23, 2013 (**B, E**); and May 23, 2014 (**C, F**). Different letters above bars indicate significant differences between treatments (*P*< 0.05, n = 3). Bars indicate standard deviation.

Compared to those in CK, the plants in the RG and WC treatments showed significantly increased plant height; however, MG only increased plant height at the first sampling time and not at later time points (Fig 1). RG, WC, and MG treatments also increased trunk diameter at the first two sampling times; at the last time point, the trunk diameters increased in RG and WC treatments but not in MG (Fig 1). Repeated measures ANOVA analysis showed that the three mulching treatments significantly affected plant height and trunk diameter (Table 3), suggesting that soil mulching stimulates plant growth.

The maximum plant height and trunk diameter were 59.2 ± 1.3 cm and 0.91 ± 0.2 cm, respectively, in the WC treatment (Fig 1C and 1F). Mulching with wood chips significantly improved plant growth, likely though improving soil properties (e.g., moisture, SAN, and SOM) and plant physiological parameters. Scharenbroch [43] also reported that organic material could be beneficial for tree establishment, weed control, and root decay. However, Iles and Dosmann [14] found that tree height and stem diameter of red maple trees (*Acer rubrum* L.) were not affected after two years of mulching with organic materials; although organic mulches are known to remarkably influence soil temperature, moisture, and pH. Ferrini et al. [46] also found that mulching with pine bark did not significantly affect the height or trunk diameter of ornamental trees. These contradictory findings might be attributed to the fact that organic matter content differs across different soil layers, with the 0–10 cm soil layer having more positive impacts on shoot growth and physiological attributes of plants, and > 15 cm soil layers suffer from decreased water penetration [43], increased soil tension, reduced shoot growth, and enhanced plant stress [47].

Plants of the RG treatment were significantly taller (Fig 1) and this finding is consistent with that of Fairbourn [9], who suggested that gravel mulch could increase crop yield by improving the interaction between the increased soil water content and soil temperature. Holloway [48] also found that five woody plant species grew better after stone mulch treatments than after other mulch treatments. However, Iles and Dosmann [14] found that the height and stem diameter of red maple were not affected after two years of mulching with crushed red brick, pea gravel, lava rock, carmel rock, and river rock.

In this study, mulching with MG increased soil moisture and SOM content, thereby stimulating plant growth (Fig 1, first sampling time); however, this effect decreased with time as shown by the non-significant increase in plant growth at the second and third time points. This might be attributed to the fact that manila turf grass competed with ‘Rixianggui’ for nutrients and water, leading to nutritional deficiencies in the mulched plants [1]. In fact, SAN was lower in MG than in CK, although this difference was not significant at the second and third time points (Tables 1 and 2). Previous studies show that tree establishment and growth are inhibited by turf grass mulches such as Bermuda grass, tall fescue, and Kentucky bluegrass [17]. Watson [11] also reported that, compared to plants grown in bare soil, root density was remarkably reduced in plants mulched with grass. Hence, mulching with turf grass might not be beneficial for plant growth in the long term.

## Conclusions

Our findings suggest that round gravel, wood chips, and manila turf grass help create a healthy soil environment and that different mulches have different effects on the soil properties at the two soil depths that were sampled. Soil moisture and SOM increased at both soil depths, whereas mulching had no effect on the bulk density, pH, or STN. SAN increased in soils mulched with round gravel and wood chips, but not with manila turf grass mulch. Root activity, soluble sugar, and chlorophyll a contents increased in all the mulched soils, especially those mulched with round gravel and wood chips. Plant height and trunk diameter were significantly higher after mulching with round gravel and wood chips; however, the stimulating effect of manila turf grass decreased gradually because of the competition for SAN between turf grass and ‘Rixianggui’. Therefore, considering the effect of mulching on soil properties and plant growth and physiology, round gravel and wood chips are a better choice than manila turf grass in ‘Rixianggui’ nurseries and plantations. Further studies are required to determine the effects of mulch quality and mulch-layer thickness on shoot and root growth.

## Supporting Information

S1 Certificate Certificate Of English Editing. (PDF)
